# Development of Immunodetection Systems Using a Specific Antibody Against the Recombinant Coat Protein for Detecting Sugarcane Streak Mosaic Virus

**DOI:** 10.3390/pathogens14111106

**Published:** 2025-10-30

**Authors:** Intan Ria Neliana, Bambang Sugiharto, Rikno Harmoko, Wahyu Indra Duwi Fanata

**Affiliations:** 1 Postgraduate Program in Biotechnology, University of Jember, Jl. Kalimantan No. 37, Kampus Tegalboto, Jember 68121, Indonesia; nelianaintanria@gmail.com; 2 Department of Agricultural Product Technology, Faculty of Agricultural Technology, University of Jember, Jl. Kalimantan No. 37, Kampus Tegalboto, Jember 68121, Indonesia; 3 Laboratory of Molecular Biology and Biotechnology, Center for Development of Advanced Science and Technology, University of Jember, Jl. Kalimantan No. 37, Kampus Tegalboto, Jember 68121, Indonesia; 4 Center for Genetic Engineering, National Research and Innovation Agency, Jl. Raya Jakarta-Bogor, Cibinong, Bogor 16911, Indonesia; rknharmoko@gmail.com; 5 Doctoral Program in Agrultural Sciences, Faculty of Agriculture, University of Jember, Jl. Kalimantan No. 37, Kampus Tegalboto, Jember 68121, Indonesia; wahyuindra.faperta@unej.ac.id

**Keywords:** sugarcane, SCSMV, coat protein, antibody, immunoblot, ELISA, immunocapture RT-PCR

## Abstract

Sugarcane streak mosaic virus (SCSMV) is one of the mosaic viruses found in mixed infection with two or more viruses. Infections of mosaic viruses show highly similar mosaic symptoms and are difficult to distinguish. This study aimed to develop a specific antibody against SCSMV that could potentially be utilized to differentiate mosaic virus infections. The cDNA encoding the coat protein (CP) of SCSMV was isolated by RT-PCR from the symptomatic sugarcane leaves. The cDNA was then used for the production of CP for the development of its polyclonal antibody. The nucleotide sequence of the cDNA showed high homology of 94.2–97.3% at the amino acid level with the *CP*-cDNA of SCSMV isolated from India (AM749403.1), Africa (OR195142.1), and USA (U75456.1). CP was produced as a recombinant protein with a molecular size of 36.5 kDa in *Escherichia coli*. The injection of recombinant CP into a rabbit resulted in the production of polyclonal antibodies, which were used for the immunodetection of SCSMV in sugarcane. Immunoblot analysis revealed a specific reaction of SCSMV-CP in symptomatic sugarcane leaves. ELISA (Enzyme-Linked Immunosorbent Assay) and IC-RT-PCR (Immunocapture-Reverse Transcription-Polymerase Chain Reaction) using the CP antibody proved successful for detecting SCSMV infection in sugarcane leaves. The results indicate that the SCSMV-CP antibody is suitable for an immunodetection system and exhibits high specificity for SCSMV infection.

## 1. Introduction

Sugarcane (*Saccharum* spp.) is an important crop that supplies almost 80% of the world’s sugar production [1]. The growth and productivity of sugarcane are affected by environmental stresses, including climate change, pests, and diseases caused by fungi, bacteria, and viruses [2]. Sugarcane mosaic virus is a major pathogen in sugarcane that exhibits a distinctive mosaic pattern on the leaves, leading to reduced growth and productivity of sugarcane. In Indonesia, the mosaic symptom was found in all sugarcane plantations, with disease incidence and severity of 78% and 65%, respectively [3]. The mosaic disease is caused by plant viruses, such as sugarcane streak mosaic virus (SCSMV), sugarcane mosaic virus (SCMV), and sorghum mosaic virus (SrMV), which are difficult to distinguish by their symptoms. Moreover, a recent study found that a multiple mosaic virus infection caused the mosaic disease, and SCSMV was identified as a major mosaic virus in sugarcane [4].

SCSMV is a flexuous filamentous virus and typically has a size of 890 nm long and 15 nm wide, with a monopartite ssRNA genome, approximately 10 kb in length [5]. The SCSMV genome contains a single long open reading frame (ORF), which encodes a polyprotein that includes a coat protein (CP) located at the 3′-end terminus [6]. Gene for CP was intensively applied for facilitating detection of the plant viruses by serological methods, such as for the detection of infection of the cucurbit yellow stunting disorder virus [7], pelargonium zonate spot virus [8], cucumber mosaic virus [9], alfalfa mosaic virus [10], and SCMV [11]. Moreover, coat-protein-mediated resistance has been studied to induce resistance against SCMV [12] and rice [13]. The RNAi (RNA interference) mediated resistance was also applied to induce resistance against the mosaic virus in plants, such as in tomato [14], soybean [15], and sugarcane [16]. Numerous studies have exploited the viral *CP* gene isolated from various viruses for their potential in multiple applications.

Antibodies against viral CP are a valuable tool for detecting and studying viruses. The antibody was traditionally prepared using purified virions as immunogen; however, this preparation has limitations, including the requirement for virus propagation, a low antibody titer, and a lengthy process [17]. However, the development of antibodies against recombinant proteins was commonly used for immunodetection and has several advantages, such as a shorter production process and a highly specific detection probe in research and immunodiagnostics, although the production involved genetic engineering techniques [18,19]. Recombinant DNA technology is often used to produce antibodies for various immunoassays, such as ELISA, immunoblot, immunofluorescence, and immunocapture-PCR, to identify and quantify viruses.

This study aimed to develop immunodetection systems using a recombinant CP for producing polyclonal antibodies against SCSMV. The *CP* gene was cloned using RT-PCR and used for the production of recombinant CP to elicit a polyclonal antibody in a rabbit. The antibody was applied for the detection of SCSMV in sugarcane by immunoblot, immunocapture-PCR, and ELISA methods. The antibody demonstrated high specificity in detecting SCSMV and did not cross-react with other mosaic viruses.

## 2. Materials and Methods

### 2.1. Cloning and Plasmid Construction for the Recombinant Coat Protein of SCSMV

Sugarcane cultivar PS-881 leaves infected with mosaic virus, exhibiting yellowing and chlorosis symptoms, were used for the isolation of *SCSMV-CP* cDNA. The leaves were ground with liquid nitrogen, and total RNA was isolated using a RNAprep Pure Plant Plus Kit (Tiangen, Beijing, China) as described previously [4]. Total RNA (1 µg) was converted to cDNA using a ReverTra AceTM Kit (Toyobo, Osaka, Japan), followed by PCR amplification of cDNA for CP-SCSMV using a KOD-Plus-Neo Kit (Toyobo, Osaka, Japan) and a pair of primer forward F1: 5′-YTGGTGGAGYAAGCAYACAGGAAARG-3′ and reverse R1: 5′-TCTCACTGGTCTGAAACAGGGTGAGC-3′. The PCR reaction was performed at 95 °C for 5 min for pre-denaturation, followed by 35 cycles of denaturation at 95 °C for 30 s; annealing at 52 °C for 30 s, and extension at 72 °C for 1 min, then followed by a final extension at 72 °C for 5 min using a T100TM Thermal Cycler (Bio-Rad, Hercules, CA, USA). The PCR product was visualised using a GelDoc system (Major Science, Saratoga, CA, USA), purified using a GenepHlow Gel/PCR Kit (Geneaid, Taipei, Taiwan), and used for nucleotide sequence determination.

To obtain the recombinant coat protein, the *SCSMV-CP* cDNA was amplified using PCR with pairs of primers F2: 5′-TTGCATATGGGGGGAGAAGCACTC-3′ and R1: 5′-TCTCACTGGTCTGAAACAGGGTGAGC-3′. The PCR product was purified and ligated into cloning vector pTA2 (Toyobo, Osaka, Japan), followed by transformation into *Escherichia coli* strain DH10B (ThermoFisher Scientific, Waltham, MA, USA). The pTA2 plasmid carrying *SCSMV-CP* cDNA was digested with restriction enzymes *Nde*I and *EcoR*I and subcloned into the expression vector pET28a containing a fusion hexa-histidine (6-His) tag at the N-terminal (Invitrogen, Carlsbad, CA, USA). The ligated product was transformed into *E. coli* BL21 (DE3) (Thermo Fisher Scientific, Waltham, MA, USA). The colony PCR with the F2 and R1 primer pairs, and enzymatic digestion using *Nde*I and *EcoR*I, were used to determine the bacterial transformants.

### 2.2. Production and Purification of the Recombinant CP-SCSMV

The recombinant protein was produced by growing the *E. coli* BL21 transformant carrying the pET28-SCSMV-CP construct in 3000 mL of LB (Luria–Bertani) medium containing 50 μg/mL kanamycin at 37 °C overnight on a shaking incubator (150 rpm). The expression of the *SCSMV-CP* was induced by adding IPTG (Isopropyl β-D-Thiogalactoside) at 0.5 mM, and the bacterial cell was harvested by centrifugation at 6000× *g* for 10 min at 4 °C. The recombinant protein SCSMV-CP was extracted using an extraction buffer containing 50 mM NaH_2_PO_4_ and 300 mM NaCl (pH 8). Soluble protein was separated from insoluble protein by centrifugation at 12,000× *g* for 10 min at 4 °C. The insoluble fraction was resuspended in a solubilization buffer containing 50 mM NaH_2_PO_4_, 300 mM NaCl, and 8 M Urea (pH 8). The recombinant SCSMV-CP was purified using Ni-NTA (Nickel-Nitrilotriacetic acid) affinity resin, as previously reported [11]. The purified protein was further separated by SDS-PAGE (Sodium Dodecyl Sulfate Polyacrylamide Gel Electrophoresis) (12.5% acrylamide), and the corresponding SCSMV-CP band was excised and eluted from the gel by electroelution. The purified protein was dialyzed against phosphate-buffered saline at 4 °C overnight. The protein purity was evaluated using SDS-PAGE, and the protein concentration was determined with Bradford reagent.

### 2.3. Development of Polyclonal Antibody

The purified protein recombinant SCSMV-CP (500 µg) was mixed with 500 µL of Freund’s complete adjuvant at a ratio of 1:1 (*v*/*v*) and injected subcutaneously into a male New Zealand White rabbit (6 weeks old). The antiserum collected before the first injection was identified as pre-immune (P1). Two weeks after the first immunization, the rabbits were boosted with five additional subcutaneous injections of 250 µg of recombinant protein, mixed with 500 µL of Freund’s incomplete adjuvant at a 1:1 (*v*/*v*) ratio, administered every week. The immune response to CP immunization was monitored by collecting antiserum following boosting injections and performing Ouchterlony double diffusion analysis using pre-immunized serum as a control.

### 2.4. Immunoblot Analysis

To determine the sensitivity of the serum after immunization, a series of serial dilutions of the purified SCSMV-CP (0.01, 0.05, 0.1, 0.2 μg) was separated using SDS-PAGE and then transferred onto PVDF transfer membranes (ThermoFisher Scientific, Waltham, MA, USA) using a semi-dry transfer blotter (Biorad, Hercules, CA, USA). The membrane was reacted with SCSMV-CP antibody diluted in Tris-Buffer Saline (TBS) with 0.5% skim milk (1:3000) overnight with gentle agitation. After three washes with TBS, the membrane was incubated with the secondary antibody, goat anti-rabbit IgG alkaline phosphatase-conjugate (AB_11180336) (Thermo Fisher Scientific, Waltham, MA, USA), for 60 min. The corresponding protein bands were stained with the BCIP (5-bromo-4-chloro-3-indolyl phosphate) and NBT (4-bromo-2-para-nitro blue tetrazolium chloride) substrate (Wako, Osaka, Japan).

Healthy and symptomatic sugarcane leaves with a single SCSMV infection and a double SCSMV-SCMV infection were used for immunoblotting analysis. The leaves were extracted using an extraction buffer containing 50 mM Tris-HCl (pH 7.5), 1 mM EDTA, 5 mM dithiothreitol (DTT), and 10% polyvinylpolypyrrolidone (PVP). After centrifugation at 12,000× *g* for 10 min at 4 °C, the soluble proteins were separated and stored in a deep freezer. The insoluble proteins were extracted from the debris using a buffer containing 50 mM Tris-HCl (pH 7.5), 1 mM EDTA, 2% SDS, and 30% sucrose. Approximately 20 μg of the soluble and insoluble proteins were separated by SDS-PAGE and then subjected to immunoblot analysis using antibodies against SCSMV-CP and SCMV-CP for the double infection of SCSMV-SCMV.

### 2.5. Indirect ELISA (Enzyme-Linked Immunosorbent Assay) Analysis

The indirect ELISA analysis was carried out as described in a recent study [20] using an ELISA kit (ThermoFisher Scientific, Waltham, MA, USA). The sensitivity of the antibody was evaluated using serial dilutions of the recombinant SCSMV-CP at 0.001, 0.005, 0.01, 0.05, and 0.1 µg/mL. The determination of SCSMV infection was performed using the crude protein extract from infected SCSMV and healthy sugarcane leaves, diluted at concentrations of 0, 0.3, 0.5, 1, and 2 µg/mL. The diluted proteins and negative control buffer (containing 50 mM NaH_2_PO_4_ and 300 mM NaCl) were mixed with 50 mM carbonate buffer, pH 9.4, and coated onto 96-well microtiter plates. After overnight incubation at 4 °C, the wells were washed three times with phosphate-buffered saline containing 0.05% Tween 20 and then blocked with blocking buffer for 1 h at 37 °C. Following three washes, the wells were incubated with 100 µL of serially diluted sera (1:1000, 1:3000, 1:5000, and 1:10,000) for 2 h at 37 °C. The secondary antibody, horseradish peroxidase (HRP)-conjugated goat anti-rabbit IgG (ThermoFisher Scientific, Waltham, MA, USA), was diluted to 1:1500 and 1:3000 and incubated for 2 h at 37 °C. The wells were rewashed, and then 100 µL of a substrate solution containing 3,3′,5,5′-tetramethylbenzidine was added. They were incubated for 30 min at room temperature. The reaction was terminated using a stop solution containing 0.16 M H_2_SO_4_, and the absorbance was measured with a spectrophotometer at 450 nm. A sample was considered positive when the OD450 ratio between the positive (P) and negative (N) samples was above 2.1 [21,22].

### 2.6. Reverse Transcriptase-PCR (RT-PCR) Analysis

RT-PCR analysis was conducted to detect SCSMV infection in sugarcane leaves using a pair of primers, forward F1—reverse R1, and total RNA isolated from healthy and symptomatic sugarcane leaves, followed by PCR amplification as described in the above section [4]. The amplified DNA products were separated by gel electrophoresis (1% agarose) and visualized using a GelDoc system (Major Science, Saratoga, CA, USA).

### 2.7. Immunocapture Reverse Transcriptase PCR (IC-RT-PCR) Analysis

Immunocapture (IC) was performed using the coating and capture methods previously reported [23,24]. The antibody against SCSMV-CP was coated in a microtube by adding 50 µL of diluted antibody (1:500, 1:1000, and 1:1500) in coating buffer for 2 h at 4 °C. After incubation, the non-bound antibody was eliminated from the microtube by washing three times with PBS containing 0.05% (*v*/*v*) Tween 20. The mosaic virus was extracted from healthy and symptomatic sugarcane leaves with varying symptom levels using 3 volumes (*w*/*v*) of PBS buffer containing 0.05% Tween 20, 1% Na_2_SO_3_, and 2% PVP. A 50 µL of the prepared sample was added to each of the coated tubes and incubated overnight at 4 °C. After three washes with PBST buffer, the virus’s immunological capture was applied to synthesize cDNA. The RT-PCR analysis was performed using a primer pair designed to detect the *SCSMV-CP* gene. The amplified cDNA band was then separated by agarose gel electrophoresis and visualized using a GelDoc system (Major Science, Saratoga, CA, USA).

## 3. Results

### 3.1. Cloning of SCSMV-CP cDNA

**Figure 1:**
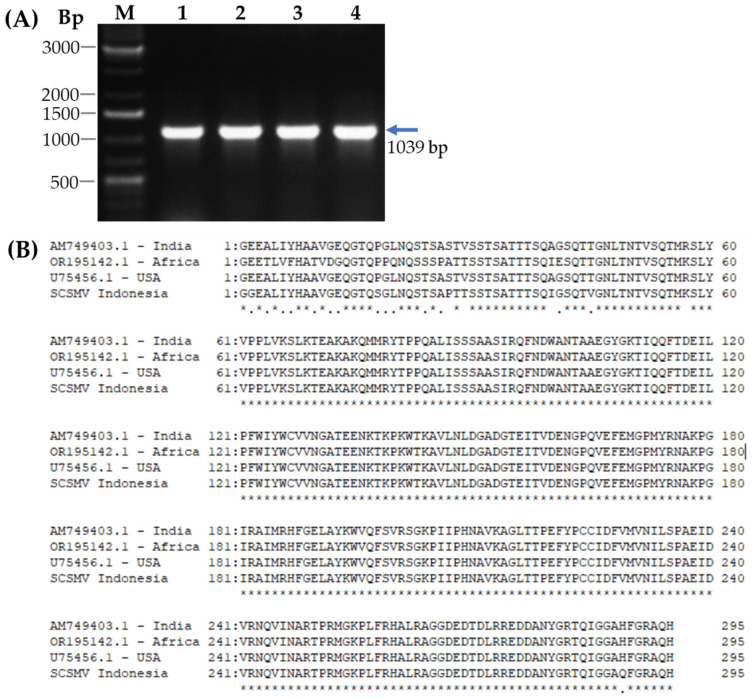
Cloning of cDNA for CP-SCSMV from mosaic-symptomatic sugarcane leaves by RT-PCR. (**A**) Separation of the cDNA by RT-PCR analysis in agarose gel electrophoresis. (**B**) Comparison of the deduced amino acid sequence for SCSMV-CP-cDNA sequence from Indonesia, India, Africa, and the USA. Lanes 1, 2, 3, and 4 are cDNA from four different symptomatic sugarcane leaves (arrow). M, DNA Ladder 1 kb.

Cloning of the cDNA encoding the coat protein (CP) of SCSMV was conducted using RT-PCR with total RNA isolated from mosaic symptomatic sugarcane leaves. The RT-PCR analysis showed a single band of cDNA in agarose gel electrophoresis (Figure 1A). The sequence determination revealed that the cDNA has a molecular size of 1039 bp, encoding coat protein containing 296 amino acids (Supplementary Figure S1). Homology search revealed that the cDNA has high homology (94.24–97.29%) with the SCSMV-CP cDNA isolated from India (AM749403.1), Africa (OR195142.1), and USA (U75456.1) at the amino acid level (Figure 1B). In addition, comparison of the amino acid sequences deduced from cDNAs showed that the SCSMV-CP differed almost entirely from the amino acid sequence of CP-SCMV [11] (Supplementary Figure S2). These results indicated that the cDNA for SCSMV-CP was successfully isolated and named *SCSMV-CP* cDNA PS-881 Jember isolate.

### 3.2. Expression and Purification of Recombinant SCSMV-CP

**Figure 2:**
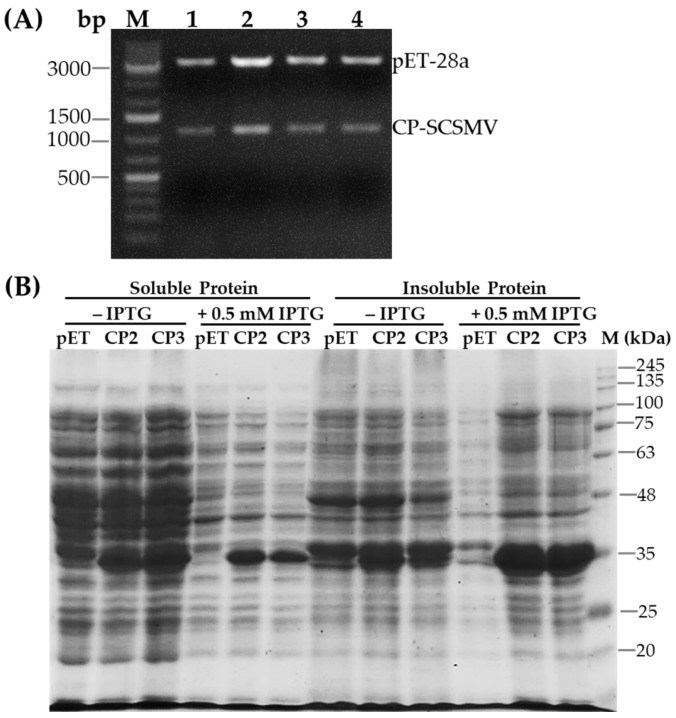
Expression of cDNA for CP-SCSMV in *E. coli* BL21. (**A**) Separation of digested pET28a-CP SCSMV DNA using *Nde*I and *EcoR*I in agarose gel electrophoresis. Lanes 1, 2, 3, and 4 are DNA digested from four different DNA constructs. M, a marker of DNA Ladder 1 kb. (**B**) Protein profile of the *E. coli* extracts containing the pET-28a CP-SCSMV construct with and without 0.5 mM IPTG induction. The pET, CP2, CP3 represent *E. coli* containing pET28a plasmid, isolate 2 and isolate 3 of *E. coli* containing pET28a-CP SCSMV, respectively. M, Protein ladder.

To produce recombinant CP, the pTA2 plasmid carrying SCSMV-CP cDNA was digested with restriction enzymes *Nde*I and *EcoR*I and ligated into the expression vector pET28a (Supplementary Figure S3). The DNA construct was subsequently transformed into *E. coli* BL21, and the restriction enzyme digestion revealed the SCSMV-CP cDNA insert (Figure 2A). Production of the recombinant CP was performed by culturing *E. coli* BL21 in liquid media containing the corresponding antibiotic overnight. The protein profile extracted from the bacterial cells was visualized using SDS-PAGE. The results showed that the recombinant CP, with a molecular size of 36.2 kDa, was more highly concentrated in insoluble protein than in soluble protein. The addition of 0.5 mM IPTG induced a pronounced expression of the recombinant CP (Figure 2B). Furthermore, the molecular size of the CP of SCSMV, at 36.2 kDa, was slightly higher than the predicted size using Expasy (https://web.expasy.org/translate/ accessed on 15 January 2025), which was 34.6 kDa.

**Figure 3:**
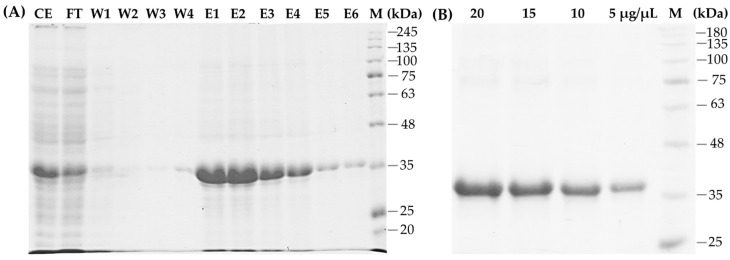
Protein separation of purified recombinant CP-SCSMV using SDS-PAGE. (**A**) The CP-SCSMV was purified using NiNTA resin and separated using SDS-PAGE. Lines CE, FT, W1–W4, and E1–E6 represent crude extract, flow through, washing unbound protein, and bound protein fraction at 250 mM imidazole, respectively. (**B**) Visualisation of purified recombinant CP after electroelution by SDS-PAGE at concentrations of 5, 10, 15, and 20 µg. M, marker of protein ladder.

Purification of the recombinant protein was conducted using an affinity resin of NiNTA. Separation with SDS-PAGE showed that the recombinant CP (36.2 kDa) was eluted by the addition of 250 mM imidazole (Figure 3A). However, small amounts of protein were also found in the flow-through (FT) before elution, which was caused by excessive protein loading of the resin. The collected recombinant CP was further separated by SDS-PAGE, excised, and eluted from the gel. As expected, a highly pure recombinant CP in a total amount of almost 2 g was isolated (Figure 3B) and used as an antigen for the development of polyclonal antibodies.

### 3.3. Development of the Polyclonal Antibody Against SCSMV-CP

**Figure 4:**
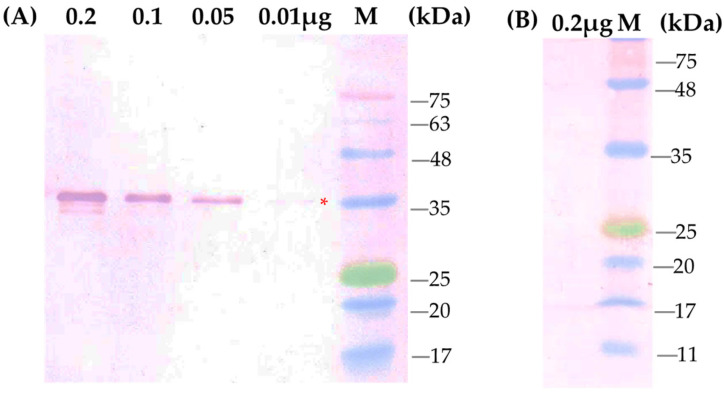
Immunoblot analysis of recombinant protein CP-SCSMV using the polyclonal antibody. (**A**) Serial concentrations of purified recombinant CP-SCMV were separated by SDS-PAGE and subsequent immunoblot analysis using the polyclonal antibody, and (**B**) using pre-immune serum. M, marker of protein ladder. Red asterisk shows the band of recombinant protein CP-SCSMV.

The polyclonal antibody was developed through subcutaneous injection in a New Zealand White rabbit. Observation of the raised rabbit antiserum using the Ouchterlony test revealed that the antibody response became visible after the second booster injection and did not appear in the pre-immunized serum (Supplementary Figure S4). The antiserum was collected after the second to fifth booster and used for immunodetection of SCSMV infection. The sensitivity of the antibody was evaluated by immunoblot analysis using the purified SCSMV-CP. The antibody clearly reacted with the SCSMV-CP at a low concentration of 10 ng of the recombinant protein (Figure 4A). In contrast, the pre-immunized serum did not react with the protein (Figure 4B).

### 3.4. Detection of SCSMV Infection in Sugarcane Leaves

#### 3.4.1. Immunoblotting Analysis

**Figure 5:**
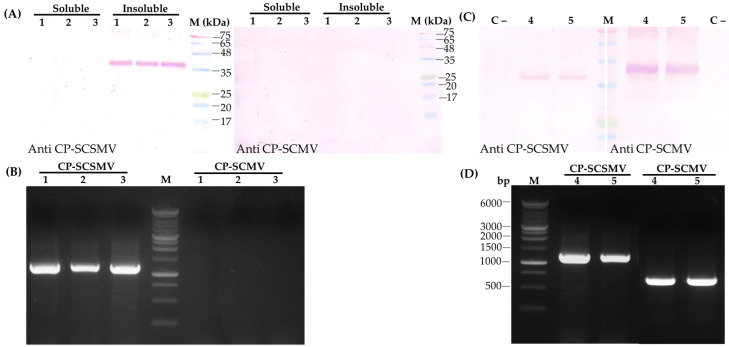
Detection of SCSMV in symptomatic sugarcane leaves by immunoblot and RT-PCR analysis. (**A**) Immunoblot analysis using the antibody against SCSMV-CP. (**B**) Detection of SCSMV and SCMV by RT-PCR analysis. Lines 1, 2, and 3 represent the SCSMV-infected sugarcane leaves. (**C**) Immunoblot analysis using an antibody against SCSMV-CP (left) and SCMV (right). Lines C-, 4, and 5 represent healthy sugarcane leaves, and the co-infection of SCSMV and SCMV, respectively. (**D**) Detection of co-infection of SCSMV and SCMV by RT-PCR analysis. Lines 4 and 5, co-infection of SCSMV and SCMV. M, Protein ladder and DNA Ladder 1 kb.

The effectiveness of the antibody against SCSMV-CP was evaluated using immunoblot, ELISA, and immunocapture RT-PCR (IC-RT-PCR) analysis. The immunoblot analysis demonstrated that the antibody reacted with insoluble protein but not with soluble protein isolated from symptomatic sugarcane leaves, with a molecular size of 36.2 kDa. (Figure 5A). Furthermore, the effectiveness of the antibody was further evaluated using the protein extracted from double-infection sugarcane leaves with SCMV and SCSMV. The immunoblot was conducted separately with the antibody against SCMV-CP generated previously [11] and SCSMV-CP in this study. Interestingly, the antibodies against SCMV-CP and SCSMV-CP reacted with the specific corresponding CPs, which differ in molecular size at 36.2 kDa and 36.7 kDa, respectively (Figure 5B). RT-PCR analysis using specific primer pairs for SCSMV and SCMV detection confirmed the co-infection of SCSMV and SCMV (Figure 5C). However, the molecular size of the DNA of SCSMV generated from RT-PCR analysis was higher than that of SCMV because the primer pair used in the analysis for SCSMV detection covered the 3′-UTR (Supplementary Figure S1). These results clearly indicate that the SCSMV-CP antibody has high specificity and does not react with the CP of SCMV.

#### 3.4.2. Indirect ELISA Analysis

The sensitivity of the antibody was further examined with ELISA analysis using the pure recombinant SCSMV-CP. The increasing antigen protein concentration (0–0.1 µg/mL) increased absorbance at 450 nm. However, dilution of the antibody concentration from 1000 to 10,000 times, as well as the second antibody (horseradish peroxidase conjugate), from 1500 to 3000 times, decreased the absorbance (Supplementary Table S1). These results show that 0.001 µg/mL of antigen protein is the minimum concentration detected by the antibody through indirect ELISA. Moreover, diluting the antibody 1000 times resulted in the highest absorbance at 450 nm, which is then used for further ELISA analysis.

**Figure 6:**
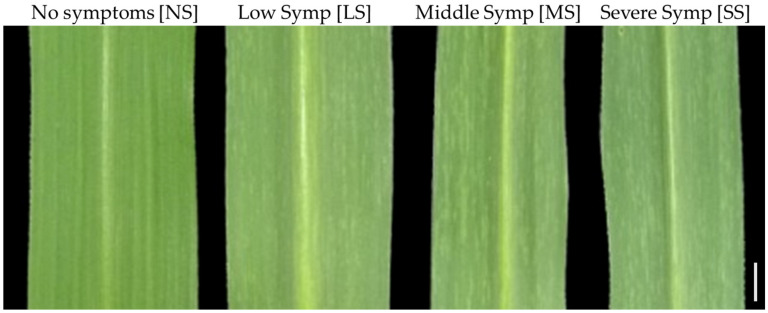
Photograph of healthy and various levels of symptomatic sugarcane leaves. NS, No symptoms; LS, Low symptoms; MS, middle symptoms, and SS, severe symptoms. Scale bar  =  1 cm.

The optimal working dilution of the antibody, 1000 times, was used to detect SCSMV infection in sugarcane leaves. ELISA analysis was performed using healthy and symptomatic sugarcane leaves with varying symptom severities, ranging from low to medium to severe infection (Figure 6). The results showed that healthy (mosaic symptom-free) leaves indicate lower absorbance at 450 nm compared to symptomatic leaves at all dilution levels of the crude extract proteins (Table 1). As expected, the higher protein concentrations, ranging from 0.3 to 2 µg/mL, resulted in increased absorbances in all symptomatic leaves. Furthermore, P/N ratio analysis was employed to differentiate between positive and negative virus infections. The ratio of absorbance between measured samples and the buffer determined the P/N ratio. An absorbance ratio less than 2.1 is considered uninfected, and a greater absorbance ratio is considered infected by the virus [20]. The P/N analysis revealed that healthy sugarcane leaves have a P/N ratio of less than 2.0, while symptomatic leaves have a P/N ratio of more than 2.1 at all protein dilution levels. The P/N ratio was found to be greater in severe symptomatic compared to lower symptomatic sugarcane leaves.

#### 3.4.3. Immunocapture Reverse Transcription PCR Analysis

**Figure 7:**
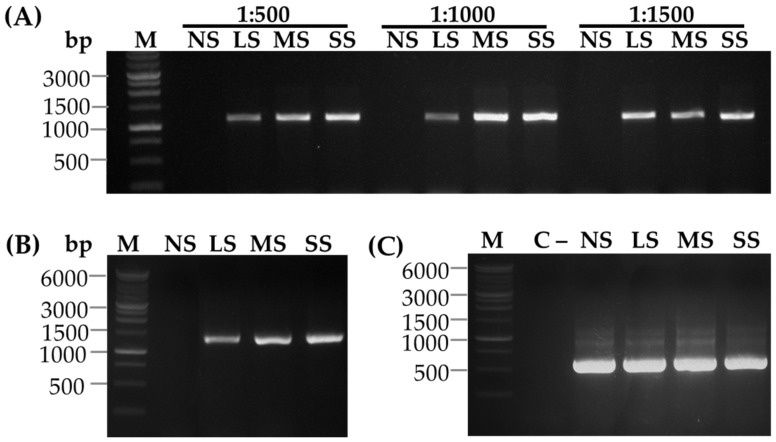
Detection of SCSMV infection in healthy and symptomatic sugarcane leaves using IC-RT-PCR and RT-PCR analysis. (**A**) IC-RT-PCR analysis of healthy and symptomatic sugarcane leaves. NS, LS, MS, and SS represent no symptoms, low symptoms, middle symptoms, and severe symptoms, respectively. (**B**) RT-PCR analysis for detection of SCSMV infection, and (**C**) Actin expression. C- water as negative control; M, DNA Ladder 1 kb.

Immunocapture Reverse Transcription PCR (IC-RT-PCR) is a sensitive and robust molecular diagnostic method used to detect and identify RNA viruses by combining the specificity of antibody-based viral capture with the RT-PCR for amplifying viral RNA. The detection of SCSMV with IC-RT-PCR resulted in the amplification of a single DNA band with a molecular size of 1039 bp in symptomatic sugarcane leaves. However, the corresponding DNA band was not found in the healthy sugarcane leaf (Figure 7A). The dilution of the SCSMV-CP antibody 500- and 1000-fold resulted in a similar pattern of DNA band intensities, which increased in proportion to the severity of SCSMV infection, ranging from low (LS) to severe symptoms (SS). However, the corresponding DNA bands were not correlated with the degree of infection at a 1500-fold dilution. Similarly, molecular sizes and band intensities of the DNA were also observed when the viral infection was detected by RT-PCR analysis (Figure 7B). In addition, the *Actin* DNA bands were at the same level in all sugarcane leaves, regardless of the degree of SCSMV infection (Figure 7C). This result indicates that the antibody at a 1000-fold dilution is suitable for IC-RT-PCR analysis.

## 4. Discussion

SCSMV is one of the most important sugarcane diseases, and the infection leads to the manifestation of mosaic-like symptoms, which develop irregular patterns of light and dark green or yellow patches or streaks. Mixed infections with two or more plant viruses have naturally occurred in sugarcane and are challenging to differentiate. This mixed mosaic virus infection alters photosynthetic gene expression [4] and reduces the growth and productivity of sugarcane [25]. The development of an antibody against SCSMV is a crucial strategy for identifying the causative agent in mosaic disease. The high specificity of the antibody against recombinant SCSMC-CP was successfully developed and used for immunodetection of mosaic disease in sugarcane. Immunoblot analysis using the antibody revealed that the antibody was able to differentiate the infection between SCSMV and SCMV (Figure 4). Further analysis confirmed that the antibody against SCSMV-CP can be applied for the SCSMV infection in sugarcane using ELISA (Table 1) and IC-RT PCR analysis (Figure 5). These results indicate that the antibody against recombinant SCSMC-CP is effective in differentiating the mosaic virus infection in sugarcane.

Recently, next-generation sequencing (NGS) has provided robust and rapid identification of potential diseases caused by viruses, compared to the molecular and immunological methods [26]. However, the NGS approach requires complex equipment and bioinformatics capabilities [27]. Virus detection using immunological systems, such as immunoblot, ELISA, and IC-RT-PCR, is widely employed with high accuracy. Although these methods accurately detect virus infections, they are not suitable for rapid detection in sugarcane fields. The methods require laboratory facilities and lengthy analysis times. Development of a rapid diagnostic test based on the antibody offers several advantages, including fast and convenient operation, as well as low cost, as demonstrated by the rapid detection of the Sacbrood virus in *Apis cerana* [28], and cucumber green mottle mosaic virus [29]. The antibody developed against recombinant SCSMV-CP was effective for detecting SCSMV and was able to differentiate between mosaic virus infections. The availability of this antibody facilitates the screening of sugarcane in sanitation programs and the development of sugarcane resistant to SCSMV.

The use of a resistant sugarcane cultivar is considered the most effective approach to managing viral disease. The overexpression of viral protein, as well as RNAi technologies, has been applied to engineered sugarcane cultivars having resistance to the SCMV [12,16]. Comparison of the methods revealed that the RNAi approach was more effective in producing a higher resistance against the mosaic virus. The mixed infection between SCSMV and SCMV with synergistic interaction has been found and caused a reduction in photosynthetic gene expression that might exacerbate yield in sugarcane [4]. The development of antibodies against SCMV [11], and SCSMV will support the genetic engineering of sugarcane cultivars resistant to mosaic diseases. We are undertaking an experiment to develop transgenic sugarcane with enhanced resistance to SCMV and SCCMV infections. Availability of these antibodies will help to differentiate the infection of both SCMV and SCSMV.

## 5. Conclusions

The specific antibody against CP-SCSMV has been developed using a recombinant protein of CP-SCCMV. The developed antibody was then used for immunodetection of SCSMV infection in sugarcane. Immunoblot analysis revealed that the antibody exhibits high specificity and does not react with the CP of SCMV. Further immunodetection using ELISA and IC-RT-PCR methods showed that the antibody has sensitivity to differentiate the degree of SCSMV infection. These results indicate that the antibody against recombinant SCSMC-CP is effective in determining the mosaic virus infection in sugarcane.

## Figures and Tables

**Table 1:** Determination of SCSMV infection in healthy and various levels of symptomatic sugarcane leaves using indirect ELISA analysis.

Sugarcane Leaves Samples	Coating Protein Concentration μg/mL	Absorbance of Buffer Extraction *Abs*450	Absorbance of Crude Extract Protein *Abs*450	P/N Ratio
No Symptom [NS]	2	0.125	0.218	1.74
	1	0.106	0.177	1.66
	0.5	0.089	0.126	1.43
	0.3	0.072	0.106	1.46
Low Symptomatic [LS]	2	0.125	0.663	5.31
	1	0.106	0.447	4.20
	0.5	0.089	0.215	2.43
	0.3	0.072	0.156	2.16
Middle Symptomatic [MS]	2	0.125	0.721	5.77
	1	0.106	0.473	4.45
	0.5	0.089	0.253	2.86
	0.3	0.072	0.168	2.33
Severe Symptomatic [SS]	2	0.125	0.734	5.87
	1	0.106	0.486	4.57
	0.5	0.089	0.286	3.23
	0.3	0.072	0.204	2.82

## Data Availability

The original contributions presented in this study are included in the article. Further inquiries can be directed to the corresponding author.

## Supplementary Materials

The following supporting information can be downloaded at: https://www.mdpi.com/article/10.3390/pathogens14111106/s1, Figure S1: Nucleic acid sequence and amino acid sequence translated from the cDNA of CP-SCSMV; Figure S2: Comparison of the amino acid sequences deduced from cDNAs of CP-SCMV (Darsono et al., 2018) [11] and CP-SCSMV (this study); Figure S3: The map of the CP-SCSMV cDNA in the pET28a expression vector containing 6xHis tag (Invitrogen); Figure S4:Double immunodiffusion of the Ouchterlony test; Table S1: Optimization of indirect ELISA with various concentrations of antigen and antibodies.
