# Supplementary material for: Development of Immunodetection Systems Using a Specific Antibody Against the Recombinant Coat Protein for Detecting Sugarcane Streak Mosaic Virus
Source: Pathogens. 2025 Oct 30;14(11):1106. doi: 10.3390/pathogens14111106 (PMC12655272; doi:10.3390/pathogens14111106)
Supplement: Supplementary file 1 [file pathogens-14-01106-s001.zip › pathogens-3930577-supplementary.pdf]

Supplementary Figure S1. Nucleic acid sequence and amino acid sequence translated from the cDNA of CP-SCSMV. The amino acid sequence was determined using Genetyx software and an asterisk at the position of 885 nucleic acid sequence is the stop codon.

```
   1 GGGGGAGAAGCACTCATCTATCACGCCGCAGTTGGAGAGCAAGGAACACAATCTGGGTTA  60
     G  G  E  A  L  I  Y  H  A  A  V  G  E  Q  G  T  Q  S  G  L
  61 AATCAAAGCACATCGGCCCCAACGACATCGAGTACGTCAGCTACGACAACAAGCCAAATA  120
     N  Q  S  T  S  A  P  T  T  S  S  T  S  A  T  T  T  S  Q  I
 121 GGAAGTCAAACAGTAGGAAACTTAACCAACACAGTCTCACAAACGATGAAATCTTTATAC  180
     G  S  Q  T  V  G  N  L  T  N  T  V  S  Q  T  M  K  S  L  Y
 181 GTTCCACCGCTGGTTAAGTCACTCAAAACGGAGGCCAAGGCAAAGCAGATGATGCGATAC  240
     V  P  P  L  V  K  S  L  K  T  E  A  K  A  K  Q  M  M  R  Y
 241 ACACCACCACAAGCCCTCATTTCATCATCAGCAGCATCAATACGACAATTCAATGATTGG  300
     T  P  P  Q  A  L  I  S  S  S  A  A  S  I  R  Q  F  N  D  W
 301 GCGAACACAGCAGCTGAAGGGTATGGAAAAACTATTCAACAGTTTACAGATGAGATACTC  360
     A  N  T  A  A  E  G  Y  G  K  T  I  Q  Q  F  T  D  E  I  L
 361 CCTTTTTGGATCTATTGGTGTGTCGTTAATGGAGCAACTGAAGAAAACAAGACGAAGCCG  420
     P  F  W  I  Y  W  C  V  V  N  G  A  T  E  E  N  K  T  K  P
 421 AAGTGGACGAAAGCTGTGCTAAACCTAGACGGAGCAGATGGTACAGAGATCACTGTAGAC  480
     K  W  T  K  A  V  L  N  L  D  G  A  D  G  T  E  I  T  V  D
 481 GAAAACGGACCCCAAGTGGAGTTTGAAATGGGGCCAATGTACAGAAACGCCAAACCTGGT  540
     E  N  G  P  Q  V  E  F  E  M  G  P  M  Y  R  N  A  K  P  G
 541 ATTCGCGCGATTATGAGACATTTTGGCGAATTGGCGTATAAGTGGGTTCAGTTCTCGGTT  600
     I  R  A  I  M  R  H  F  G  E  L  A  Y  K  W  V  Q  F  S  V
 601 CGTAGCGGGAAACCCATAATACCACACAATGCAGTGAAGGCAGGATTAACCACACCAGAG  660
     R  S  G  K  P  I  I  P  H  N  A  V  K  A  G  L  T  T  P  E
 661 TTTTATCCTTGCTGCATTGATTTCGTGATGGTGAATATCCTCTCACCAGCAGAAATAGAC  720
     F  Y  P  C  C  I  D  F  V  M  V  N  I  L  S  P  A  E  I  D
 721 GTGCGTAACCAGGTGATTAACGCACGTACACCCCGGATGGGAAAGCCTTTATTCCGTCAT  780
     V  R  N  Q  V  I  N  A  R  T  P  R  M  G  K  P  L  F  R  H
 781 GCTCTTAGAGCCGGGGGAGATGAGGACACGGACCTGCGTAGGGAAGATGATGCAAACTAT  840
     A  L  R  A  G  G  D  E  D  T  D  L  R  R  E  D  D  A  N  Y
 841 GGAAGGACGCAGATCGGTGGCGCTCAATTTGGGCGCGCCCAGCACTGATTTTCAGTGCAG  900
     G  R  T  Q  I  G  G  A  Q  F  G  R  A  Q  H  *
 901 TTATCCATATATTATCGTATCTACGTATCAACTGGTTCTCTTGGATAGGCTGATGGTAGT  960

 961 TGCCTGACCGCTTAGACGATCATCAGGAGCTACTAGTGGATCATTCGGAGATTGCTCACC  1020

1021CTGTTTCAGACCAGTGAGA  1039
```

Supplementary Figure S2. Comparison of the amino acid sequences deduced from cDNAs of CP-SCMV (Darsono et al., 2018) [11] and CP-SCSMV (this study).

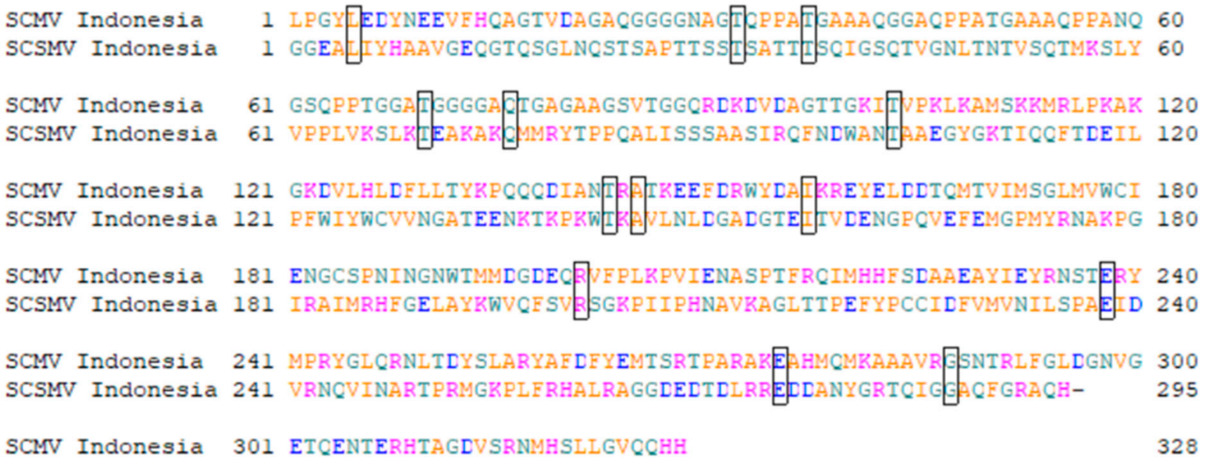

Supplementary Figure S3. The map of the *CP-SCSMV* cDNA in the pET28a expression vector containing 6xHis tag (Invitrogen). The CP-SCMV cDNA was inserted *Nde*I and *Eco*RI by subcloning the cDNA from pTA2 plasmid.

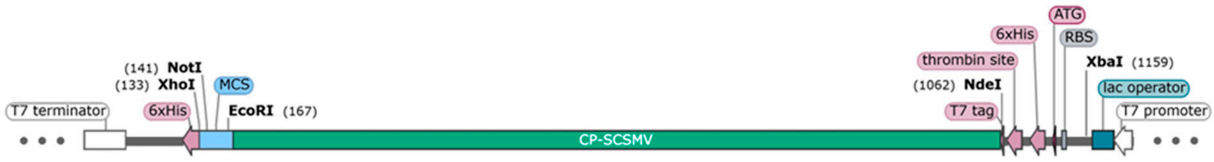

Supplementary Figure S4. Double immunodiffusion of the Ouchterlony test. The pre-immunised antiserum (P1), and antiserum after booster 2 (S1), 3 (S2), 4 (S3), 5 (S4), 6 (S5) weeks were reacted with the antigen of pure recombinant CP through the double diffusion test, and the binding precipitates antigen-antibody were stained with CBB.

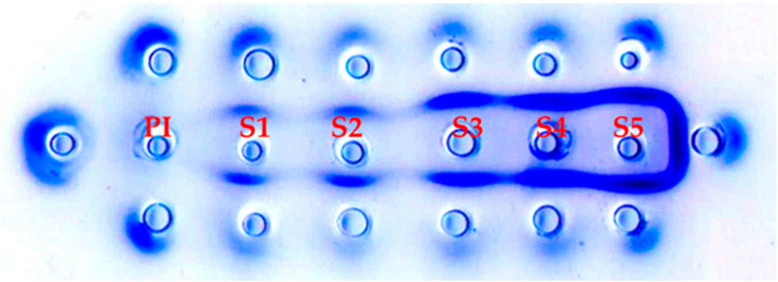

Supplementary Table S1. Optimization of indirect ELISA with various concentrations of antigen and antibodies.

Coating antigen concentration μg/mL	Conjugated Antibody Dilution	P/N of the Optical Density (OD) Reading at Different Levels of the Serum Dilution			
		1000	3000	5000	10000
0.001	1500	2.63	2.36	2.36	2.27
0.005		10.12	7.82	7.62	6.11
0.01		15.79	14.67	13.72	12.81
0.05		33.37	31.11	30.25	27.90
0.1		35.03	33.56	31.95	29.69
0.001	3000	2.28	2.20	1.95	1.79
0.005		7.49	4.94	4.70	4.24
0.01		13.86	11.91	8.79	7.20
0.05		28.35	26.52	26.41	23.86
0.1		26.68	26.71	26.07	26.63
